# Pandemic Influenza Vaccines: What did We Learn from the 2009 Pandemic and are We Better Prepared Now?

**DOI:** 10.3390/vaccines8020211

**Published:** 2020-05-07

**Authors:** Steven Rockman, Karen Laurie, Ian Barr

**Affiliations:** 1 Seqirus, 63 Poplar Road, Parkville 3052, Victoria, Australia; Steve.Rockman@seqirus.com (S.R.); karen.laurie@seqirus.com (K.L.); 2 Department of Microbiology and Immunology, The University of Melbourne at the Peter Doherty Institute of Infection and Immunity, 792 Elizabeth Street, Melbourne 3000, Victoria, Australia; 3 WHO Collaborating Centre for Reference and Research on Influenza, at the Peter Doherty Institute for Infection and Immunity, 792 Elizabeth Street, Melbourne 3000, Victoria, Australia

**Keywords:** influenza vaccine, pandemic, pandemic preparedness, influenza, A(H1N1)pdm09, seasonal influenza vaccine, pandemic influenza vaccine, pandemic vaccine

## Abstract

In 2009, a novel A(H1N1) influenza virus emerged with rapid human-to-human spread and caused the first pandemic of the 21st century. Although this pandemic was considered mild compared to the previous pandemics of the 20^th^ century, there was still extensive disease and death. This virus replaced the previous A(H1N1) and continues to circulate today as a seasonal virus. It is well established that vaccines are the most effective method to alleviate the mortality and morbidity associated with influenza virus infections, but the 2009 A(H1N1) influenza pandemic, like all significant infectious disease outbreaks, presented its own unique set of problems with vaccine supply and demand. This manuscript describes the issues that confronted governments, international agencies and industries in developing a well-matched vaccine in 2009, and identifies the key improvements and remaining challenges facing the world as the next influenza pandemic inevitably approaches.

## 1. Introduction

Influenza remains a viral respiratory disease of global importance. In 2018–2019, the world observed the centenary of the start of the 1918–1919 influenza pandemic. The impact of this pandemic was an estimated toll of 50 million deaths resulting in an observable decline in life expectancy in many countries [1,2]. Subsequent pandemics occurred in 1957–1958 and 1968–1969 during periods when significant progress in medical science had been made, including the identification of the viral agent, influenza, the development of influenza vaccines, antiviral drugs and diagnostics and, importantly, antibiotics to treat secondary bacterial infections [3]. In 2009, the first pandemic of the 21st century occurred with the emergence of a new form of the A(H1N1) virus, now known as A(H1N1)pdm09. In the first year A(H1N1)pdm09 circulated, it was estimated to have been responsible for between 151,700 and 575,400 deaths [4]. The virus mainly infected young children and adults but also caused significant deaths in the elderly (over 65 years of age) population [5]. Ten years on, we review some of the lessons learned from the 2009 pandemic in regard to influenza vaccines and their production, what has changed and what challenges remain when the next, inevitable, influenza pandemic occurs.

## 2. Global Capacity of Influenza Vaccine Production

**Figure 1:**
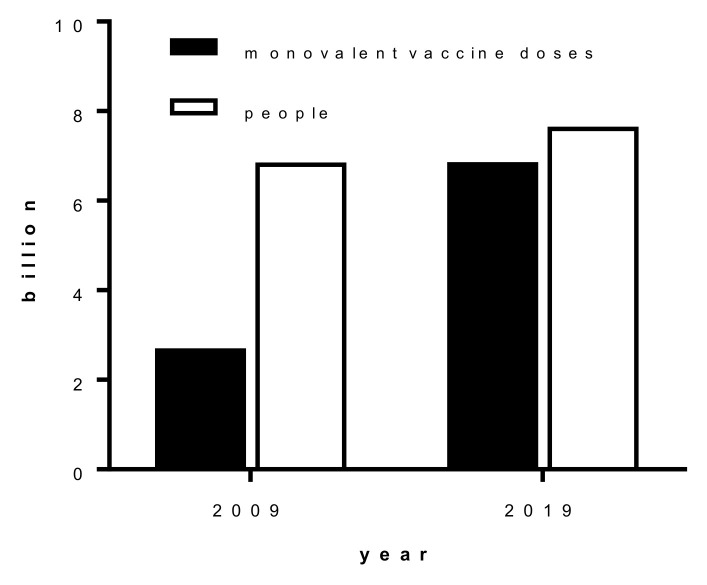
Comparison of estimated global vaccine capacity (15 μg HA/dose, black bars) and global population (white bars) in 2009 and 2019 (estimated from [6,7,8]).

**Figure 2:**
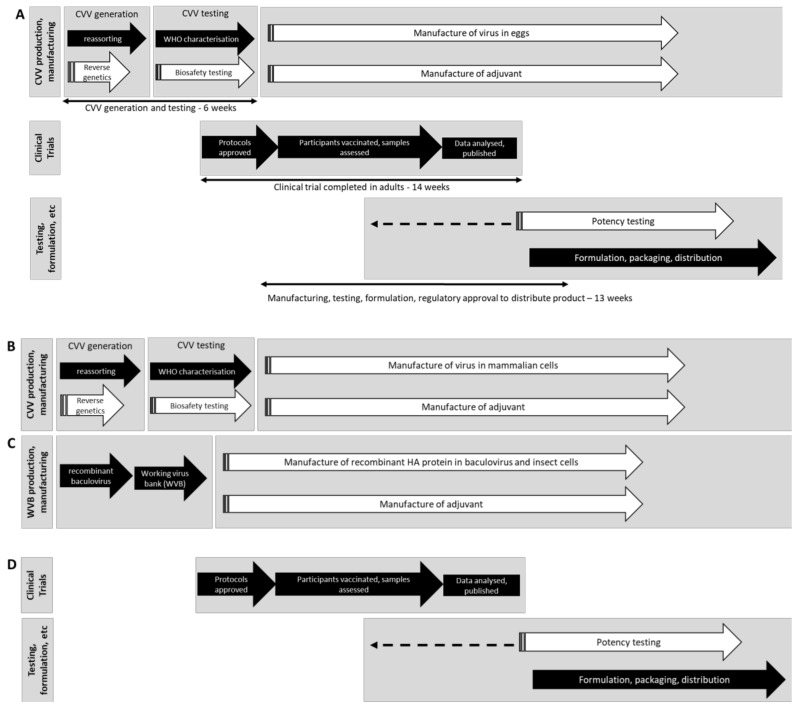
Processes for the manufacturing of influenza virus vaccines. The manufacturing process for influenza vaccines with steps where improvements have been realised since 2009 are shown in white (adapted from [23].) (**A**) In 2009, the majority of influenza vaccine was manufactured in embryonated hen’s eggs. Candidate vaccine viruses (CVVs) were prepared by reassorting in 2009 and now there is improved capability for more rapid reverse genetics. CVVs are characterised at a WHO Collaborating Centre (CC) to ensure matched antigenicity and HA/NA sequences to the recommended strain. Strains were tested to ensure attenuation by multiple methods in 2009, and now this has been streamlined through risk assessment. Clinical trials may be performed to understand the immunogenicity of a novel vaccine. A vaccine is formulated based on potency testing; new assays may enable potency to be assigned earlier. Some vaccines are formulated with adjuvants, enabling dose sparing. Vaccines are packaged and distributed. The timeline for each step in 2009 is indicated [23]. (**B**) Manufacture of influenza vaccine in mammalian cells requires a CVV that is either prepared in eggs (as in **A**) or cells [26]. All CVVs are characterised at a WHO CC and are also assessed to ensure attenuation, prior to large-scale manufacturing. (**C**) The HA gene is inserted into the baculovirus genome and the resulting recombinant baculovirus is amplified in insect cells to create a working virus bank (WVB). The WVB is used for full-scale manufacture. Creation of a WVB is more rapid than generation and testing of CVVs. (**D**) Clinical trials may be performed with manufactured product from the cell culture or recombinant protein systems. Product is formulated and tested for potency prior to release (dotted line indicates that potency testing occurs once assays are developed).

In 2009, the global influenza monovalent pandemic vaccine capacity was 2.7 billion doses (assuming a single 15 μg haemagglutinin (HA)/dose [6]) and the world’s population was approximately 6.85 billion, meaning approximately one-third of the world could be immunised with a pandemic influenza vaccine. In 2019 it has been estimated that, under the same assumptions, the global influenza pandemic vaccine capacity has increased to 6.4 billion doses, meaning that there would now be a pandemic vaccine for over three-quarters of the global population [7,8] (Figure 1). Various factors have contributed to this increase in manufacturing capability, such as: the World Health Organization (WHO) Global Action Plan for Influenza Vaccines (GAP) Program, a coordinated effort to strengthen vaccine production capability [9,10]; the switch from trivalent to quadrivalent seasonal vaccines; the use of high dose antigen (x4 the seasonal antigen dose) for seasonal vaccines for the elderly; and the increased use of adjuvants in vaccines [8]. Thus, the level of pandemic influenza vaccine coverage has substantially increased despite the growth in global population. One large caveat on these estimates is that they represent a full year’s output of manufacturing. Full-scale vaccine manufacturing of a pandemic vaccine would only commence once a suitable manufacturing strain was prepared and tested, which together takes approximately 3–6 months (see below and Figure 2), meaning that a vaccine would only be available after the first wave of the pandemic had passed through many countries.

## 3. Currently Available Influenza Vaccines

In 2009, over 95% of the registered influenza vaccines in the world were generated in embryonated hen’s eggs and at least two-thirds of influenza vaccines were produced by just seven manufacturers [11]. This included split virion and sub-unit vaccines, as well as live attenuated intranasal vaccines [11]. Since 2009, the number of influenza vaccine manufacturers has significantly increased (currently at least 32 manufacturers with full-scale manufacturing facilities and licensed vaccines) and manufacturing is more widespread, particularly in Asia and South America ([11,12,13,14], E. Sparrow personal communication). Furthermore, a number of new technologies have been developed or expanded and are now registered for use in producing influenza vaccines. These include the increased use of mammalian cells to grow viruses, and the further use of baculovirus and insect cell expression systems as well as other expression systems such as plant-based vaccine production systems that are also in late stage development [15,16]. Most of these systems are now being utilised for seasonal influenza vaccines but could be rapidly re-deployed to produce a pandemic influenza vaccine when required. Moreover, vaccines produced using nanoparticles and chimeric HAs have been recently used in phase 1 clinical trials and have potential to be used in the context of a pandemic [17,18,19]. There are key differences in the manufacturing processes of egg, cell and recombinant protein systems to ensure that the maximum number of doses for each platform are produced (Figure 2). Egg-based influenza vaccines require the development of high growth reassortants to improve virus yield, a process which involves either reassorting candidate vaccine viruses (CVV) with high growth laboratory strains of influenza, or the use of “reverse-genetics” (RG), whereby influenza viruses are produced “synthetically” using a series of plasmids encoding the gene segments of the pandemic virus and the high growth laboratory strain [20]. Both activities occur in just a handful of specialised laboratories. These reassorted/RG CVVs must undergo antigenic analysis, sequencing of the HA and neuraminidase (NA) genes and preliminary yield evaluation prior to large-scale production. Our experience from generating vaccines over many decades, including for the pandemics of 1957, 1968 and 2009 and for pre-pandemic strains such as A(H5N1) and A(H7N9), is that the yield of vaccine available is determined by the specific strain. Cell culture production systems, such as those that use various forms of MDCK or Vero cells, allow for faster scale-up in case of pandemics, as they are not limited by the supply of embryonated hen’s eggs. Wildtype CVVs may be used if the pandemic virus is of low pathogenicity and the virus is high yielding in cell-based production, otherwise CVVs generated by reverse genetics may be required. Cell culture vaccines may also offer an advantage with certain influenza viruses by providing antigenically better matched vaccines to some influenza strains in humans by avoiding egg adaptions, which can reduce vaccine effectiveness [21]. Vaccines that utilise conventional recombinant protein technologies have the advantage of not requiring the pandemic virus or a CVV for production and can instead be produced with just the sequence of the HA (and other genes such as the NA or matrix if required). This approach requires a suitable expression system for the particular system in use (such as baculovirus, E. coli, mammalian or plant cells) for manufacture. This is estimated to take 2–3 weeks to produce [22], which is 3–4 weeks quicker than the generation and testing of a reassorted CVV for egg vaccine manufacture in 2009 [23] (Figure 2). Even with the advent of these new technologies since 2009, the testing and downstream process requirements align most of these products with respect to vaccine availability. Currently, egg-based influenza vaccines still constitute around 85%–90% of doses given annually, and so progress away from egg manufacturing of influenza vaccines has been and is likely to continue to be slow. This may be enhanced by the recent announcement of a US Presidential Executive order made on 19 September 2019 that “directs actions to reduce the United States’ reliance on egg-based influenza vaccine production……” among other measures to enhance the US preparedness for influenza pandemics (see [24]).

## 4. Challenges That Influenza Vaccine Manufacturers Encountered in 2009 and the Key Changes That have Been Made Since

Key challenges are detailed below and summarised in Table 1.

### 4.1. Biocontainment

At the beginning of the 2009 pandemic, the initial requirement for biocontainment of the wildtype A(H1N1)pdm09 virus was under Biosafety Level (BSL)-3 conditions as directed by the WHO [25]. This presented a significant issue in the preparation of CVVs as both wildtype and reassortant viruses (made by classical reassorting processes or by RG), required the use of BSL-3 facilities. Furthermore, it was deemed that attenuated CVVs (see below) were needed to ensure the safety of staff that may be exposed during vaccine manufacture as the majority of the large vaccine manufacturing plants could not operate as BSL-3 (only one influenza vaccine plant had BSL-3 capability in operation globally in 2009). Thus, the CVVs required safety testing (including ferret pathogenicity testing) to ensure attenuation prior to release for large-scale manufacture. In 2009, two reassorted attenuated CVVs of the A(H1N1)pdm09 virus that had passed safety testing, IVR-153 (from CSL) and X-179A (from NYMC), were available to manufacturers by the end of May.

Safety testing of attenuation of influenza A strain remains a requirement for all newly developed influenza A pandemic strains in 2020. Building on experiences with (1) A(H1N1)pdm09 virus, (2) the attenuation of highly pathogenic avian influenza viruses and (3) emergence of low pathogenic avian influenza viruses infecting humans (e.g., A(H7N9)), the WHO convened two informal consultations to re-visit the guidelines (TRS 941, Annex 5, [27]) for the safe development and production of vaccines for human pandemic influenza viruses and viruses with pandemic potential in 2017 and 2018 [28]. The WHO now recommends that the containment conditions for the generation or manufacture of a pandemic, or potential pandemic CVV, is determined by both the category of CVV (reassortant, wildtype, subtype) and an activity-based risk assessment. This risk assessment takes into account the laboratory or manufacturing activity, the facility, PPE available and environmental risks [28]. While safety testing remains a requirement for all pandemic CVVs, a CVV may be shipped prior to completion of some or all tests if deemed acceptable on the basis of additional risk assessments. Further, a CVV can be considered for large-scale manufacture prior to completion of some or all tests if deemed acceptable by the national regulatory authority [28]. Importantly, CVVs with an identical sequence to those that have been previously generated and have passed safety testing can undergo a risk assessment to determine whether re-testing is required. These changes in the guidelines allow for the faster generation of a CVV and subsequent availability of a CVV to manufacturers, particularly when the distribution of a CVV may be impeded, such as if borders are closed in future influenza pandemics.

### 4.2. Development of RG and Cell Culture CVVs

In 2009, RG strategies were undertaken by key laboratories throughout the world to develop a CVV suitable for influenza virus vaccine manufacture. The advantage of RG is the ability to (1) modify a gene sequence, such as removal of the polybasic site of a highly pathogenic H5 or H7 virus, and (2) rescue a virus where the donor backbone is considered attenuated, such as the case with the A/Puerto Rico/8/34 (A/PR/8/34), A/Ann Arbor/6/60 or A/Leningrad/134/17/57 ‘backbone’ viruses that are currently utilised in egg reassorting approaches for both inactivated and live-attenuated influenza vaccines. The introduction of a modified cleavage site has consistently reduced the pathogenicity for avian embryos and poultry [29] and the use of internal genes from backbone donor viruses has a low probability to harm human health, with many years of safe use in the manufacture of seasonal influenza vaccines [30,31]. Notably, attenuation is not only required for safety, but to also enable manufacturing of highly pathogenic avian influenza viruses that are lethal to chicken embryos. Fortunately, the A(H1N1)pdm09 virus did not have a polybasic cleavage site like some H5 and H7 viruses and so the manipulation of the HA gene by RG was not required.

At the time of the 2009 pandemic, the generation of reassortants by RG was possible by cloning the HA and NA genes from RNA extracted from the wildtype virus along with the other genes from A/PR/8/34. Hence, the requirement for biocontainment due to handling of a wildtype virus and the time taken for cloning, including site-directed mutagenesis and confirmation of the removal of wildtype virus, delayed generation of a CVV by RG. Furthermore, initial RG rescue attempts of an early A(H1N1)pdm09 strain, A/California/04/2009, were unsuccessful when the rescued virus was passaged in embryonated eggs in preparation for vaccine manufacture. It was later found that rescue could be rectified upon introduction of the egg adaptation mutations K209T and Q223R in the HA gene that were observed later in the egg reassortant virus (14). Host passage adaptation highlights a significant problem that may occur with novel viruses when using the RG rescue method and while these problems may be overcome eventually, this may cause significant delays in CVV availability.

Since 2009, two approaches have been developed for the rapid generation of CVVs which require the modification of the wildtype sequence for manufacture. Firstly, rescue of influenza virus by RG has been improved by the use of synthetically generated plasmids. Sequence modifications, such as the removal of the polybasic cleavage site in the HA protein, can be directly incorporated into the nucleotide sequence obviating the need for site-directed mutagenesis and cloning from the wildtype virus [32,33]. This approach significantly reduces the time from receipt of the synthesised DNA to the rescue of RG virus in embryonated hen’s eggs to approximately 7 days, notably shorter than the 21 days typically required to generate a conventional reassortant virus in eggs. A further technical advance that has improved the availability of CVVs is the isolation and manufacture of influenza virus in mammalian cells which have a much higher virus isolation rate than eggs [21]. However, manufacture in mammalian cells may still have similar biocontainment issues to egg-based systems with future influenza pandemics, as this system also relies on the use of a live influenza virus. This issue is avoided with the baculovirus system and other recombinant protein expression systems which only require the HA sequence to be made available for vaccine production [15]. Passage and rescue of influenza viruses in mammalian cells also negates the chance of egg adaptations as cell lines used to grow influenza virus in vitro have similar receptors as the dominant human receptors in the upper respiratory epithelium (α-2,6-linked sialosides) that binds the virus [34]. In contrast, embryonated eggs have a majority of receptors that are α-2,3-linked sialosides, which can drive egg adaptations in the HA of the virus that may result in improvement in the growth and yield of the virus [34]. However, some egg adaptations can affect the neutralising epitopes that surround the sialoside binding site of the HA protein and affect the antigenicity of the CVV and therefore the eventual vaccine, but passage in mammalian cells minimises this risk [21,35]. Egg adaptions are also avoided with the baculovirus system in which the HA protein sequence used is like the cell-propagated virus which is then expressed in insect cells [15].

### 4.3. Availability of Pre-Pandemic Stockpiles and Pre-Pandemic CVVs

Since the outbreak of sporadic human infections of highly pathogenic A(H5N1), many governments have stockpiled influenza vaccines and vaccination equipment (e.g., syringes, PPE, vials) as part of their preparedness for an influenza pandemic [36,37]. The goal of these stockpiles is to have a sufficient stock of vaccine and other items to be able to respond quickly and provide vaccines to the populations considered to be the highest priority and also to be a bridge to the availability of vaccine that will cover the remaining population. However, the available influenza pandemic vaccine stockpiles were unable to be used in 2009 as the A(H1N1) pandemic strain was unexpected and the existing stockpiles were limited to avian-origin A(H5N1) influenza vaccines (although vaccination equipment was utilised [38]). What was more readily available, at least in the Southern Hemisphere in 2009, was the 2009 seasonal inactivated influenza vaccine containing the seasonal A(H1N1) virus, that was later found to have modest efficacy against the A(H1N1)pdm09 virus (38% (95% CI: 19%, 53%)) [39].

In order to respond more quickly to a novel outbreak of influenza, laboratories that are involved in the development of CVVs, including WHO CCs, WHO Essential Regulatory Laboratories (ERLs) and reassorting laboratories, have isolated and developed, through conventional and RG techniques, a large panel of potential pandemic CVVs in order to cover as many as possible of the potential human and zoonotic influenza infections that have been observed to date [40]. To help countries select appropriate vaccines for stockpiling from this large panel, various risk assessments have also been developed by the United States Centers for Disease Control and Prevention (CDC) (known as IRAT [41]) and WHO (known as TIPRA [42]).

### 4.4. Adjuvants

An adjuvant is a biological or synthetic agent added to a vaccine to improve the immune response. An adjuvant may be included in a vaccine targeted to a population known to have a poor immune response (e.g., older adults, immunosuppressed patients). An adjuvant may also be included in a widely used vaccine to boost the immunogenicity of an antigen, enable dose sparing of antigen and induce a more rapid immune response [43,44]. These features are all favourable in an influenza pandemic situation.

In 2009, oil-in-water adjuvants MF59 and AS03 were included as part of pandemic inactivated influenza vaccines. Arepanrix (GlaxoSmithKline) and Pandemrix (GlaxoSmithKline) contained AS03 and were licensed and approved for use in Canada and Europe, respectively. Focetria (Novartis) contained MF59 and was licensed and approved for use in Europe. All of these vaccines were licensed for use in adults and children >6 months of age [45,46,47,48]. Importantly, these adjuvanted vaccines enabled dose sparing in 2009. The immunogenicity of the adjuvanted pandemic vaccines, as determined by seroconversion and seropositivity, was equivalent to that of the unadjuvanted vaccine, despite containing half or a quarter of the amount of HA antigen per dose [47]. Notably, the A(H1N1)pdm09 vaccine alone was also highly immunogenic in children and adults with one dose deemed sufficient [49,50]. This is in contrast to results found in clinical studies assessing the immunogenicity of novel avian influenza hemagglutinins, such as H5 and H7, where vaccines containing much higher levels of antigen and adjuvants, and dosing with multiple immunisations, were required for a sufficient antibody response ([51], reviewed in [52]). The addition of adjuvants to vaccines may also increase the chance of adverse events, as was observed in 2009 when narcolepsy was detected in some Swedish children following vaccination with monovalent A(H1N1)pdm09 vaccine containing the AS03 adjuvant [53,54].

Currently, four adjuvants are licensed, approved and used in seasonal inactivated influenza vaccines: MF59, Alum (Al(OH)3 and AlPO4), AS03 and virosomes (reviewed in [43,44]). It is expected that these adjuvants would also be available for inclusion in future influenza pandemic vaccines. Adjuvants can be stockpiled if they have a long shelf life (typically five years or longer), thus their manufacture would not be expected to delay vaccine release. Human trials and animal studies indicate that immunisation with A(H5N1) vaccines containing oil-in-water adjuvants increased the antibody response and was broader in cross-reactivity than immunisation with a vaccine without adjuvant ([55], reviewed in [52]). As multiple doses of vaccine may be required to mount a protective immune response against some avian influenza viruses, immunising with a stockpiled H5/H7 vaccine, with (or without) adjuvant, may be useful to prime the immune response of individuals until a matched vaccine is available ([56,57], reviewed in [52]).

### 4.5. Standardisation and Release of Vaccines

All influenza vaccines are tested for their potency to ensure that there is an appropriate amount of antigen (HA protein) present in the vaccine. The current release assay for potency, the Single Radial Immunodiffusion (SRID) assay, requires production of specific antiserum and reference antigen, followed by assay calibration by WHO ERLs, a process that takes 2–3 months to perform [58,59]. The development of antiserum is dependent upon the production of purified HA that is enzymatically cleaved from whole inactivated virus, to inject into sheep [60]. In 2009, the cleavage of HA under standard conditions was problematic due to unexpected enhanced degradation. This was encountered by multiple laboratories but NIBSC, UK produced sufficient cleaved HA to vaccinate sheep to produce antiserum and this reagent was distributed to ERLs and manufacturers worldwide without significant delay [23].

In 2010, the first workshop to discuss an alternate potency assay was convened in Ottawa, Canada. Since this time, there have been three further workshops to develop alternate approaches including ELISA, HPLC and mass spectroscopy, along with a series of publications (reviewed in [3,58]). Many of these assays are considered rapid, accurate and precise, with a greater dynamic range than the current SRID release assay. ELISA- and HA-capture-based approaches can use monoclonal antibodies that are specific and can, in principle, be applied to both a multivalent product as well as a pandemic vaccine. Pre-development of subtype-specific monoclonal antibodies can ensure rapid application of a release assay in the event of a pandemic. Alternatively, non-antibody-based assays (HPLC, mass spectroscopy) that do not require the development of specific reagents are also being considered at these workshops. These assays can determine the level of antigen but are not necessarily strain-specific nor do they assess stability [3].

## 5. What Challenges Remain for the Next Influenza Pandemic Vaccine?

### 5.1. Future Pandemic Vaccine Immunogenicity Remains an Unknown

A critical aspect of influenza vaccine development is to administer sufficient viral antigen (generally considered to be mainly HA but may also involve NA and other viral proteins) capable of inducing a partially or fully protective immune response. For seasonal influenza, the majority of individuals are primed by prior exposure via infection or vaccination and a single dose of 15μg seasonal HA antigen (per strain) in a vaccine is considered sufficient to induce protective antibody levels in healthy adults and/or reduce serious consequences of infection in the elderly [61]. In a pandemic situation, the immune status of the population is likely to be different, such as in 2009 where the young were naïve to the new strain and the elderly had been primed by prior exposure to a closely related A(H1N1) strain from the early 1900s [62,63]. In a future pandemic, the level of antigen required to induce protective immunity may differ by subtype of influenza virus. For example, to detect an antibody response following immunisation with an A(H5N1) vaccine, 90 μg of HA was required, unless the vaccine was adjuvanted, whereby 30–45 μg HA was sufficient [64]. For a novel influenza outbreak, knowledge of the antigen dose for a vaccine and the number of vaccine doses to induce a sufficiently protective response (i.e., the immunogenicity) is critical for prevention of infection and reduction in morbidity and mortality, and also determines the vaccine coverage and timing that is possible.

In 2009, manufacturers were required to conduct clinical trials in adults and children to determine antigen dose and the number of doses required for the pandemic influenza vaccine. Although this information is required for mass vaccination, this decision delayed the availability of large quantities of vaccine until November 2009 or later. While the next pandemic cannot be accurately predicted with respect to time, origin or subtype, to be best prepared, a better understanding of the immunogenicity and thus vaccine dose requirement (amount of antigen and number of doses) and the side effects profiles for novel subtypes, may reduce the need for clinical trials and ameliorate the time to vaccinate those most vulnerable, or even the overall population. Conducting pre-pandemic clinical trials in adults and children may inform the vaccination regime required to induce a sufficient immune response for a novel influenza subtype if it emerges. This would reduce the response time for the distribution of vaccine to the general population, if a similar subtype causes a future pandemic.

### 5.2. Switching Vaccine Manufacturing from Seasonal Vaccines to Pandemic Vaccine

Influenza vaccines are manufactured in dedicated facilities where a single product is produced at one time. While seasonal influenza vaccine production is virtually year-round at most sites, the outbreak of a pandemic raises the problem that manufacturers must “switch” at some stage from producing seasonal influenza vaccine to the pandemic vaccine, as both products cannot be manufactured in the same facility at the same time. The timing of this decision remains a challenge. A clear signal/trigger for vaccine manufacturers to switch vaccine production is critical to developing and distributing a pandemic vaccine rapidly to meet contractual agreements with companies and governments. Most manufacturers also have global commitments to provide seasonal influenza vaccines, and seasonal viruses may still be circulating in some locations prior to the pandemic virus arriving. The WHO declaration of a pandemic on June 11 2009 [65] provided the essential signal to “switch” for companies. This was eight weeks after the pandemic virus was first detected (15 April 2009 [66]) and seven weeks after the WHO declared a “public health emergency of international concern” (PHEIC, made on April 25 2009 [67]). In the future, if WHO is unwilling to make a unilateral declaration of an influenza pandemic, other earlier declarations by WHO such as a “PHEIC“ may be sufficient to trigger a “switch”, yet generally this decision will most likely be made by national governments in countries where manufacturers are located. Many countries (57 countries) now have pandemic plans and over 95% (55/57 countries) are triggered by the WHO declaration of a pandemic [68]. However, as only a minority of countries have local facilities to produce their own vaccine (13/57), and the majority of countries (44/57) rely on importing influenza vaccines [68], national decisions rather than an international declaration to “switch” manufacturing would make the global pandemic vaccine co-ordination difficult or impossible to achieve.

Since 2009, stakeholders have participated in three WHO “Switch” meetings to develop a global strategy and operational mechanism for initiating the pandemic vaccine response, particularly when seasonal influenza vaccine may still be needed in some parts of the world. This is a complex challenge requiring interaction and co-operation between many stakeholders including the Global Influenza Surveillance and Response System (GISRS), WHO CCs and ERLs, CVV reassorting laboratories, vaccine manufacturers, governments, clinicians and vaccine programme managers. The outcomes from these interactions are still being worked through with the aim to make this “switch” process smoother and more transparent.

### 5.3. Pandemic Surveillance

As an influenza pandemic is considered inevitable but unpredictable, the number of surveillance laboratories through the WHO has increased in the past ten years from 135 National Influenza Centres (NIC) in 105 countries at the end of 2010 [69], to at least 146 NICs in 115 countries globally in 2019 [69,70]. This has improved the ability of WHO to rapidly identify and sequence potential pandemic viruses at the earliest stage of a pandemic and allows the maximum time for CVV selection and vaccine production. High-throughput sequencing, through use of next generation sequencing technologies, more specialist influenza databases (e.g., GISAID, IRD) and online analysis tools (e.g., Nextstrain, IRD, GISAID, FluSurver) are also now available, improving surveillance capacity and understanding. There has also been a greater emphasis on the human–animal interface through the creation of the One Health concept [71] and the promotion of better co-operation between human health and animal health by WHO, World Organisation for Animal Health (OIE), Food and Agriculture Organization of the United Nations (FAO) and OFFLU (OIE–FAO global network).

### 5.4. The Nagoya Protocol and WHO Pandemic Influenza Preparedness Framework

The Nagoya Protocol on access to Genetic Resources and the Fair and Equitable Sharing of Benefits Arising from their Utilization (NP) is an international agreement which aims to share the benefits resulting from the use of a country’s genetic resources, including influenza viruses, fairly and equitably [72].

The NP treaty was enacted in October 2014 and human pathogens have been included in the legislation on NP in many countries and regions, with over 120 countries being a Party to the protocol [73]. From this point forward, the commercial use of influenza viruses (both seasonal and pandemic) sourced from a signatory country or region requires a bilateral agreement between parties (such as governments and individual manufacturers) within three months of receiving the virus. The agreement is negotiated for commercial access of an influenza virus in exchange for sharing of the benefits arising from its use, for example the provision of a set amount of pandemic influenza vaccine. Timely access to influenza viruses is critical to ensure the “best” viruses are used, so CVVs to develop vaccines are well-matched to the circulating (seasonal or pandemic) influenza viruses. In 2009, the wildtype pandemic virus was shared with influenza reassorting laboratories within days of receipt at WHO CCs to develop a CVV. However, given that the NP is now in place, there is concern that this may hinder virus sharing and consequently result in a delayed, inconsistent and reduced CVV supply, which has potential to also increase costs if not addressed. The WHO, vaccine manufacturers and governments are working to resolve this major issue.

As well as NP, another system has been introduced by WHO called the Pandemic Influenza Preparedness (PIP) framework. This was introduced on 24 May 2011 in order to increase the access in developing countries to vaccines, influenza antivirals and other pandemic-related supplies, and is supported by agreements with influenza vaccine, antiviral and diagnostic product manufacturers [74]. PIP allows the movement of potential pandemic influenza viruses within the GISRS system and to the various vaccine/antiviral/diagnostic product manufacturers. How these critical interactions will be affected by NP, or if PIP-related materials will be excluded from the requirements under the NP remains to be seen, and may not be clarified for a number of years to come.

## 6. Conclusions

The 2009 influenza pandemic, the first pandemic to occur in the post-molecular period, was generally mild in severity. Our reflection on the lessons learned from the experience of 2009 highlights significant developments, whilst also identifying ongoing challenges still present some 11 years on.

The WHO continues to expand surveillance and its ability to detect novel viruses that may result in a human pandemic. The early supply and sharing of viruses or genetic information is critical to an effective pandemic response, not only for vaccine manufacture and availability, but also for antiviral assessment and the development of diagnostics. The progress of synthetic RG and testing of backbone genes to attenuate influenza A strains have enabled the development of CVVs without the need to handle wildtype viruses and consequently reduced the biocontainment and safety testing required. Alternate potency assays hold promise to enable the determination of potency and an earlier release of vaccine than current traditional methods. Novel vaccine platforms improve the availability of vaccines. However, challenges remain for timely manufacturing of vaccines and understanding of the immunogenicity of vaccines containing novel HAs. Hence, other measures such as antivirals [75] and non-pharmaceutical interventions [76] will continue to be important components of any future influenza pandemic planning and response. Even with these challenges we are very fortunate to have systems in place for the ready production of influenza vaccines to the next pandemic threat, unlike the current pandemic threat with the novel coronavirus, SARS-CoV-2.

## Figures and Tables

**Table 1:** Status changes in the manufacture of the influenza virus vaccine from 2009–2019.

Issue	Situation in 2009	Situation in 2019
Vaccines	All vaccines (live and inactivated) prepared in embryonated hen’s eggs; only 7 manufacturers producing the majority of vaccines [11]	Expanded manufacturing platforms; embryonated hen’s eggs, mammalian cells, baculovirus, recombinant proteins; number and geographical dispersion of manufacturers increased [11]
Biocontainment	Strict biocontainment requirements for handling of wildtype virus under BSL-3 conditions by WHO	Biocontainment requirement determined locally by activity-based risk assessment [28]
	CVV generated by reassorting limited to BSL-3 laboratories	Biocontainment level required for CVV generation by reassortment determined by activity-based risk assessment [28]
	CVV generated by reverse genetics in BSL-3 laboratories, by cloning from wildtype virus	CVV generated by reverse genetics in BSL-2 laboratories using synthetic genes with any potentially pathogenic determinants (e.g., polybasic site) easily removed.
	All CVVs must undergo safety testing (including ferret testing) to demonstrate attenuation. All safety testing must be completed prior to distribution	CVV must undergo safety testing to demonstrate attenuation. Risk assessment performed to determine testing requirements prior to distribution
		Results of safety test can be used for a further CVV with identical HA/NA sequence as previously generated and tested CVV
	Manufacture can only be performed at BSL-3 with wildtype virus or BSL-2 with attenuated CVV once all safety testing is complete	Manufacture with wildtype or attenuated CVV assessed by activity-based risk assessment
Host passage adaptations	A/California/4/2009 CVV unable to be rescued in time for a potential CVV	Potential for rescue of CVV in mammalian cells as well as eggs; egg adaption sites better understood
CVVs	Emerging pre-pandemic strains under constant surveillance by WHO and panel of pre-pandemic CVVs prepared	Increased surveillance by WHO to identify emerging pre-pandemic strains, the panel of pre-pandemic CVVs is constantly updated;IRAT [41] and TIPRA [42] risk assessment tools now available to help with pandemic vaccine stockpile prioritisation
	Genetic sequence of influenza viruses limited with few specialist databases/tools and reliance on GenBank	Next generation sequencers and additional specialist influenza databases available (GISAID, IRD) and online analysis tools (Nextstrain, IRD, GISAID, FluSurver)
		Nagoya Protocol requires bilateral agreements if using virus from a signatory country
Adjuvants	MF59 and AS03 licensed, approved and used in pandemic vaccines	MF59, Alum (Al(OH)3 and AlPO4), AS03 and virosomes licensed, approved and used in seasonal vaccines. Adverse incidents to be considered before use in future pandemic
Assessment of vaccine potency and immunogenicity	SRID assay	Alternate potency assays under development
	Immunogenicity determined by clinical trial	Pre-pandemic clinical trials of novel subtypes would give insight as to appropriate dose and vaccination regime; potential for use of government stockpiles with cross-clade vaccines
‘Switching’ Manufacturing	WHO declared a pandemic on June 11 2009, resulting in ‘switch’ in manufacturing from seasonal to pandemic vaccines	Despite three “WHO Switch meetings” it is still unclear as to how a switch from seasonal to pandemic vaccines would occur; potential for ‘switch’ to be initiated by individual governments
